# Information overload, financial constraints, and psychological burdens are among the barriers faced by marginalized groups seeking curative treatments for HCC

**DOI:** 10.1097/HC9.0000000000000660

**Published:** 2025-02-26

**Authors:** Lauren D. Nephew, Courtney Moore, Nicole Garcia, Lisa Parks, Allison McKay, Alexandra T. Strauss, Sara Wiehe, Naga Chalasani, Alexandra T. Hughes-Wegner, Susan M. Rawl

**Affiliations:** 1 Department of Medicine, Division of Gastroenterology and Hepatology, Indiana University, Indianapolis, Indiana, USA; 2 Department of Community Health Partnerships, Research Jam, Community Health Partnerships, Indiana Clinical and Translational Sciences Institute, Indiana University School of Medicine, Indianapolis, Indiana, USA; 3 Department of Medicine, Division of Gastroenterology and Hepatology, Johns Hopkins University School of Medicine, Baltimore, Maryland, USA; 4 Department is Science of Nursing Care, Indiana University School of Nursing, Indiana University Simon Cancer Center, Indianapolis, Indiana, USA

**Keywords:** Black race, cirrhosis, liver transplant, social determinants of health

## Abstract

**Background:**

Patients with HCC face numerous barriers to curative therapies, particularly Black patients and those impacted by adverse social determinants of health (SDOH). This study aimed to identify patient-reported barriers and facilitators to curative therapies, to inform interventions that improve equitable access to care.

**Methods:**

We conducted 2 qualitative sessions with Black participants and participants experiencing adverse SDOH with HCC referred for liver transplant (LT) or resection. We also conducted one-on-one interviews with participants from sessions that underwent LT (n=2). Human-centered design methods, including journey mapping and group ideation, were used to identify challenges and solutions at various stages in the care pathway. Data were analyzed to identify key themes and to compare the experiences of Black patients with those experiencing adverse SDOH.

**Results:**

Both groups faced significant barriers, particularly related to information overload, communication gaps with health care providers, and the complexity of navigating the LT pathway. However, Black patients reported additional challenges related to the psychological burden of the diagnosis and distrust in the health care system, while those with adverse SDOH frequently cited financial instability, lack of social support, and challenges in coordinating care between multiple health systems. Despite these differences, common facilitators included compassionate health care teams and strong personal support networks. Both groups suggested solutions such as improvements in education timing and delivery, better communication pathways, and peer support groups to improve preparedness for treatment and recovery.

**Conclusions:**

While Black patients and those with adverse SDOH experience unique barriers, common threads—such as information gaps and desire for peer support suggest shared opportunities for interventions.

## Graphical abstract

**Figure:**
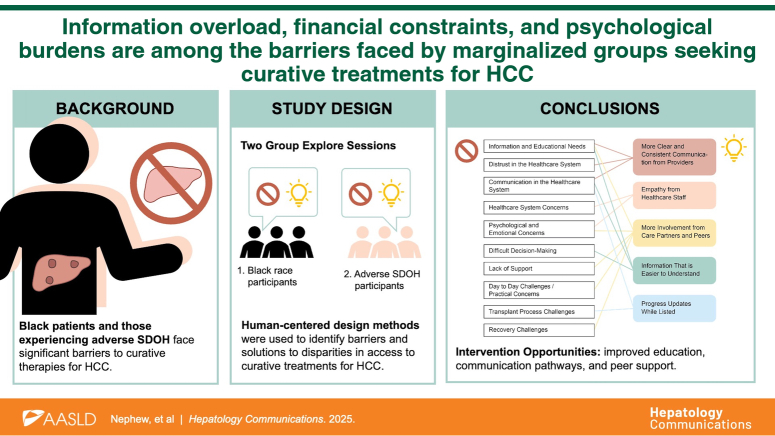

## INTRODUCTION

HCC has emerged as a significant public health concern, with its incidence in the United States contributing to over 300,000 related deaths in the past 2 decades.^1^ Surgical therapies, including resection and liver transplantation (LT), remain the most durable curative treatments for those diagnosed with early-stage disease.^2^ Yet, there are disparities in access to these therapies that disproportionately affect marginalized populations, particularly Black patients and those burdened by adverse social determinants of health (SDOH).^3^,^4^ Despite early detection and treatment advances, these groups continue to experience the lowest survival rates.^3^,^5^ This is hypothesized to, in part, be a consequence of systemic inequities that obstruct their progression through the HCC care continuum.

The SDOH, including race and socioeconomic status, are the conditions in which people live, grow, and work and have been repeatedly associated with health care access and outcomes.^6^ While the health care system increasingly recognizes the profound impact of SDOH on health outcomes, there is often a reluctance or lack of capacity to address these factors.^7^ As value-based care gains momentum, a shift toward whole-person health is driving efforts to bridge these gaps.^8^ However, the ability to capitalize on these efforts to benefit marginalized groups depends on having effective, innovative, culturally informed interventions.

Previous research has predominantly focused on health care providers’ perceived barriers to LT.^9^ This study seeks to fill that gap by exploring the lived experiences of marginalized groups pursuing curative HCC therapies. By bringing patient voices to the forefront, we aim to develop targeted interventions that can enhance health care coordination, build trust in medical institutions, and ultimately reduce disparities in treatment access and outcomes.

## METHODS

### Study design

This study employed human-centered design (HCD) methodology to explore the barriers and challenges marginalized groups face in accessing curative therapies for HCC. In HCD research, stakeholders are included as partners at every step of the intervention design process. This research method has been used by multiple disciplines to inform intervention development.^10^,^11^,^12^ To better understand care barriers and potential solutions, a group discussion is facilitated by a team of health care, research, and design professionals. While the sessions share some similarities with focus groups, they do not rely solely on asking questions in a group interview style. Instead, these sessions utilize activities to elicit participants’ tacit and latent knowledge by observing their actions, words, and creations. An additional benefit of HCD is the emphasis on bringing empathy to the process through continuous and iterative feedback from stakeholders.^13^ The HCD approach allowed us to engage participants in meaningful discussions about their experiences, utilizing journey mapping and group ideation techniques to identify unmet needs and potential solutions. The result is an intervention more closely aligned with real-world needs.

The study involved 2 group sessions, including Black participants (S1-B) and participants experiencing adverse SDOH (S2-SDOH). To supplement the data collected during these sessions, 2 individual interviews were conducted with 1 participant from each demographic group. These interviews were not intended to achieve data saturation but rather to ensure that no major themes were missing, particularly among participants who had completed the full care cascade, including LT. Given the limited number of Black participants who had reached transplantation, these individual interviews provided additional insight without seeking to draw new conclusions. The Indiana University Institutional Review Board approved the study. All participants provided written informed consent before participation.

### Participants' eligibility and enrollment

Participants were recruited from outpatient clinics within the Indiana University Health (IUH) system and Eskenazi Health between January 2023 and April 2023. Eligible participants included those at any point in the HCC-LT care pathway after referral for LT or resection, including those within 2 years of either procedure. Adults diagnosed with HCC who were considered for referral for either procedure but denied for social reasons were documented in the electronic medical record. The inclusion of patients who were dismissed before formal referral was crucial to capture the perspectives of marginalized patients who, despite being diagnosed with early-stage HCC, faced significant barriers that prevented access to cure. Participants with metastatic disease at the time of diagnosis, those over 75 years old, and those >2 years post-LT or resection were excluded from the study.

For patients meeting the clinical criteria, the electronic medical record is used to screen for key SDOH, including race, insurance type, and marital status. Participants were eligible for inclusion if they self-identified as Black, were insured by Medicaid, or were unmarried and insured by Medicare. These SDOH were selected based on findings from our statewide cohort study, which demonstrated that these factors were independently associated with increased mortality in HCC.^5^ To validate the SDOH, all participants completed a baseline survey prior to enrollment. This survey confirmed race, insurance coverage, and marital status, while also collecting additional data on annual household income and educational attainment.

### Study settings

Participants were approached for enrollment in hepatology, oncology, and interventional radiology clinics at IU Health and Eskenazi Health. At IU Health, patients are followed by a single hepatologist, consistent across interventional radiology and oncology care, and for this study, participants were recruited from a variety of clinic settings. Patients from 4 out of 5 transplant hepatology providers were ultimately included. The Indiana University Health Transplant Institute is the only LT program in the state of Indiana, performing over 189 LTs in 2023 and serving as the primary referral center for end-stage liver disease and HCC. Eskenazi Health, the safety-net hospital affiliated with the IU School of Medicine, provides a significant portion of care to minoritized and vulnerable populations in Indiana. The integration of these facilities ensured access to a diverse patient population, allowing for a comprehensive exploration of the barriers to LT for marginalized groups.

### Group sessions

**FIGURE 1:**
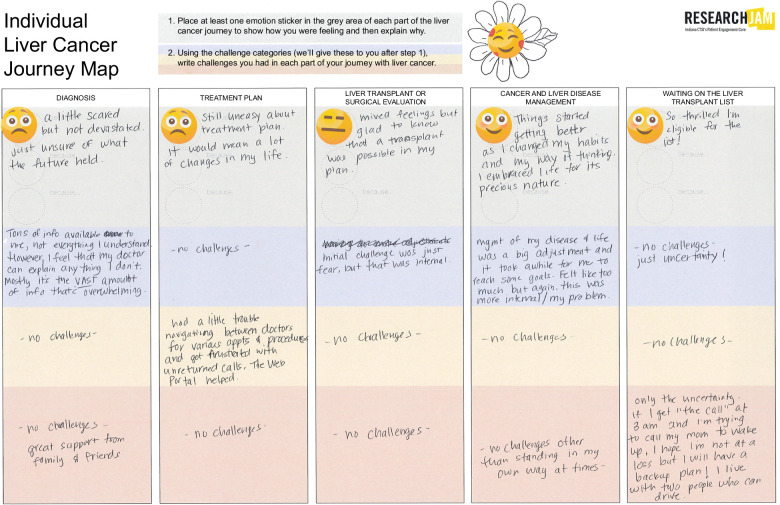
Example of individual journey map.

Group sessions were held on 2 separate evenings at IU Health. Participants were compensated for the 90-minute session with a gift card, dinner, and validated parking. For those living more than 30 minutes away, a gas card also was provided. For those without transportation, ride-share was arranged. Each session was led by a facilitator with expertise in HCD (CM). Each session was audio recorded, and study team members also took notes and photographs of activities to supplement the recordings. Examples of group activities and session questions are included in Table 1, Figure 1, and Supplemental Figure S1, http://links.lww.com/HC9/B916.

**TABLE 1:** Session questions

Primary goal/concentration	Method description and questions
Warm-up activity	• The purpose of this activity is to get to know each other a little bit and find things we have in common. • Explain the process: Introduce yourself and one thing you like to do for fun, and then someone else who also likes that thing will speak up. You will hold on to the yarn and then throw the ball to them so in the end, we will have a web of connections. • The facilitator starts to model what to do.
Individual journey mapping	The purpose of this activity is to hear your individual stories and challenges during your liver cancer journey so we can better understand what patients experience. Explain the worksheet: • Divided into 5 sections of liver cancer journey [read them]. • It’s okay if things don’t fit perfectly in a section, just put it where you think it fits best. • First, use emotion stickers to tell us how you felt during that part of the journey (or how you feel now about something that’s coming in the future if you’re not there yet). At least one sticker per section [gray section]. • *hand out challenge category sheets* • Focusing on the Diagnosis section, think about challenges you faced that had to do with information; not having the information you needed, not understanding the information you were given. Write these in the blue section. • Next, think about the challenges you had with finding what you needed in the healthcare system. For example, knowing who to call, making appointments, getting referrals. Write these in the yellow section in Diagnosis. • Next, think about Other challenges you had during Diagnosis. Write these in the red section. • Now move to the Treatment Plan section and think about challenges you had in each of the categories and then keep going through the rest of the worksheet. • If there is a part of the journey you haven’t gotten to yet or haven’t experienced, just leave that section blank.
Group journey mapping	• Ask for a volunteer to share their biggest informational challenge in Diagnosis [copy this on a blue post-it and put it on a wall map]. • Discuss and prompt: ○ Can you give an example of a time when that happened? ○ Did anyone else experience something like that? • Ask for a new volunteer, continue until 3–5 post-its in diagnosis blue. • Then move to yellow and repeat. • Then red. • Then a new section of the map and repeat. • Call on people if needed.
Group ideation	• Notify each table that they are now a team. • Instruct each team to discuss the challenges on the group journey map and choose one they want to come up with ideas for. • Ask them to write a challenge in the box on Ideation Worksheet • Introduce Worksheet: the purpose is to come up with some ways of solving your challenge during the journey. • The first step is deciding where that challenge shows up the most in the journey. Use the star stickers to show where you think that challenge comes up the most. • Choose a writer for your team—someone with good handwriting. • The next step is to come up with ideas! • Remind everyone to go for quantity over quality, no ouching, etc. Don’t judge other’s ideas or your own, all ideas are valuable. What you say can spark an idea for someone else. • Let them get out initial ideas for 7 minutes. • Then introduce “ideation prompt sheet” to help them diverge more. They can choose the prompt they want to work with and can use more than one. • Look at all your ideas, as a group, pick two that are your favorite to share with the larger group. • Have each group share their two ideas. • Gather reactions and likes from the full group [if time].

#### HCD activities

1. *Warm-up activity:* Participants took part in a “yarn connections” exercise designed to foster rapport and highlight common interests among the group. The facilitator began by holding a ball of yarn, introducing herself, and sharing a hobby. She then asked if any other participants shared a similar interest. Holding the end of the yarn, the facilitator passed the ball to the next participant, who introduced themselves and described their own hobby. This process continued until each participant held a portion of the yarn, creating a physical web of connections across the room. The activity was successful in promoting a comfortable, collaborative environment conducive to open discussion (Table 1).
2. *Individual journey mapping:* Participants completed journey map worksheets that detailed their emotions and challenges throughout their HCC journey, divided into 5 phases: Diagnosis, Treatment Planning, Liver Transplant or Surgical Evaluation, Cancer and Liver Disease Management, and Waiting on the Liver Transplant List (Figure 1 and Table 1). Emotion stickers were provided to visually express their feelings at each phase. Additionally, participants were asked to identify and record challenges within each phase, categorizing them under 1 of 4 areas: Information, Health care System, Social, or Other Needs (Supplemental Figure S1A, http://links.lww.com/HC9/B916).
3. *Group journey mapping:* Participants collectively discussed their individual challenges. The study team captured these challenges on sticky notes and placed them on a large group journey map (Supplemental Figure S1B, http://links.lww.com/HC9/B916). This visual representation highlighted common issues and provided a comprehensive view of the challenges participants faced at each stage of their journey.
4. *Group ideation:* Participants were divided into small groups to brainstorm solutions for selected challenges from the Group Journey Map (Figure 1B, and Supplemental Figure S1C, http://links.lww.com/HC9/B916). These ideas were then shared with the larger group to foster collaborative problem-solving.

### One-on-one interviews

In-person, one-on-one interviews were conducted with 2 patients who had received LT to explore specific challenges identified during the group sessions in more depth. These interviews provided additional context and details, enriching the data collected from the group activities (Supplemental Table S1, http://links.lww.com/HC9/B917). Participants were included if they had undergone LT. Otherwise, the same inclusion criteria were applied. Participants were asked the same open-ended questions used in the group discussions, such as how they felt throughout their liver cancer journey and the challenges they faced. Additionally, the interviews allowed for a deeper exploration of issues raised regarding the transplant process. The research team aimed to identify commonalities and differences in challenges, particularly focusing on the pre-evaluation and transplant process. The interviews included warm-up questions, a journey mapping activity, discussions about challenges, and more detailed questions regarding access to LT. Interviews concluded with a discussion of potential solutions to barriers identified.

### Data analysis and synthesis

Data from both the group sessions and individual interviews were de-identified and transcribed into individual sticky notes by 2 investigators (Courtney Moore and Allison McKay) using the Miro online whiteboarding software. Each note was labeled according to the source group (S1-B or S2-SDOH). The researcher team (Lauren D. Nephew, Susan M. Rawl, Courtney Moore, Allison McKay, Nicole Garcia) collaboratively organized the notes from the S2-SDOH group by thematic similarity, reaching a consensus to construct an affinity diagram with thematic headings. The same process was applied to the S1-B group, followed by the creation of a combined affinity diagram incorporating data from both S1 and S2. These themes were subsequently used to develop visual models, including concept maps illustrating the relationships between themes, and journey maps summarizing the liver cancer journey and patient-reported barriers to curative treatment. The final synthesis focused on generating visual models to represent the distinct challenges faced by Black patients and those experiencing adverse SDOH.

## RESULTS

A total of 17 participants with HCC were included in this study. The S2-SDOH group—consisting of 9 session participants and 1 individual interview—were all White, with a mean age of 64.0 years (SD±9.64). The majority of this group were men (77.8%). All participants in this group were referred for LT, and 55.6% had successfully undergone LT. The most common etiology of liver disease among this group was hepatitis C virus, accounting for 33.3% of the participants (Table 2).

**TABLE 2:** Session participant characteristics

	n (%)	n (%)	n (%)
	Adverse SDOH (n=9)	Black (n=6)	Total (n=15)
Age (y); mean, SD	64.0±9.64	62.2±8.38	63.3±8.89
MELD-3.0; mean, SD	11.33±5.20	9.83±2.14	10.73±4.20
Gender			
Male	7.0 (77.8)	5.0 (83.3)	12.0 (80.0)
Female	2.0 (22.2)	1.0 (16.7)	3.0 (20.0)
Race			
White	9.0 (100.0)	0.0 (0.0)	9.0 (60.0)
Black	0.0 (0.0)	6.0 (100.0)	6.0 (40.0)
Ethnicity			
Not Hispanic	9.0 (100.0)	6.0 (100.0)	15.0 (100.0)
Hispanic	0.0 (0.0)	0.0 (0.0)	0.0 (0)
Etiology			
MASH	2.0 (22.2)	0.0 (0.0)	2.0 (13.3)
ALD	2.0 (22.2)	0.0 (0.0)	2.0 (26.7)
HCV	3.0 (33.3)	2.0 (33.3)	5.0 (33.3)
Cryptogenic cirrhosis	1.0 (11.1)	0.0 (0.0)	1.0 (6.67)
ALD, HCV	1.0 (11.1)	3.0 (50.0)	4.0 (26.7)
HBV	0.0 (0)	1.0 (16.7)	1.0 (6.67)
Insurance			
Medicaid	3.0 (33.0)	1.0 (16.7)	3.0 (13.3)
Medicare	3.0 (33.3)	1.0 (16.7)	4.0 (26.7)
Medicaid and Medicare	3.0 (33.3)	2.0 (33.3)	5.0 (33.3)
Private insurance	0 (0.0)	1.0 (16.7)	1.0 (6.67)
Uninsured	0.0 (0.0)	1.0 (16.7)	1.0 (6.67)
Marital status			
Single	4.0 (44.4)	5.0 (83.3)	9.0 (60.0)
Married	3.0 (33.3)	1.0 (16.7)	4.0 (26.7)
Divorced	1.0 (11.1)	0.0 (0.0)	1.0 (6.67)
Widowed	1.0 (11.1)	0.0 (0.0)	1.0 (6.67)
Referred for transplant			
Yes	9 (100.0)	4.0 (66.7)	13.0 (86.7)
No	0 (0.0)	2.0 (33.3)	2.0 (13.3)
Posttransplant			
Yes	5.0 (55.6)	1.0 (16.7)	6.0 (40.0)
No	4.0 (44.4)	5.0 (83.3)	9.0 (60.0)
Milan status			
Yes	9.0 (100.0)	5.0 (83.3)	14.0 (93.3)
No	0.0 (0.0)	1.0 (16.7)	1.0 (6.67)

Abbreviations: ALD, alcohol-associated liver disease; MASH, metabolic disease–associated steatohepatitis; SDOH, social determinants of health.

The S1-B group—consisting of 6 Black session participants and 1 individual interview—had a mean age of 62.2 years (SD±8.38) and was predominantly made up of men (83.3%). Within the S1-B group, 66.7% were referred for LT, while 2 (33.3%) had not been referred due to social reasons. Only 1 participant in this group had undergone LT, while the others remained in the evaluation phase. Among this group, 50.0% had a dual diagnosis of alcohol-associated liver disease and hepatitis C virus.

Individual interviews were conducted with 2 patients who were post-LT: one was a 68-year-old Black male who was single and insured by Medicaid, and the other was a 44-year-old woman who was married and insured by Medicaid.

### Thematic analysis

Key insights and themes derived from the analysis were divided into 3 sections: (1) positive aspects of the journey, (2) barriers and challenges encountered during the care journey, and (3) solutions to care journey barriers.

### Positive aspects of the journey

#### Provider experiences

Participants consistently praised their providers, highlighting the quality of care and support they received. One participant mentioned, “My liver team, my medical team… the best. I wouldn’t trade it for anything in the world.” Another noted the personal connection with their coordinator, who went the extra mile to keep their family informed: “She wasn’t trying to second-guess me… She was trying to make sure I was covered. And I love that they took care of things.”

#### Health system experiences

Many participants expressed gratitude for the quality of care at IU Health. One patient, who relocated to Indiana for treatment, said, “I couldn’t get this treatment in [home city]. So, I’d rather be in Indianapolis, Indiana, getting treated.” Despite initial concerns about potential racial bias, another participant shared, “I thought it would be different because of the color of my skin, but everything went just fine.”

#### Social support

The importance of social support was a recurring theme. Patients spoke of the strength and reliability of their support systems, including family, friends, or community. One patient described their spouse as a crucial source of support: “She’s been cancer-free for about eight years now… And she goes through it with me. She brings me to all of my appointments.” Another participant expressed comfort in participating in the study and sharing their journey with the group, stating, “I feel great to be around [the group]… we can make a better way for everybody.”

#### Personal growth

The HCC care journey led to significant positive lifestyle changes for many participants. One patient noted, “Things started to get better as I changed my habits and my way of thinking. I embraced life for its precious nature.”

Table 3 includes additional quotes describing the positive aspects of patients’ journeys.

**TABLE 3:** Positive aspects of the journey

Theme	Supporting quotes	Attribution
Provider experiences	“The nurse was very compassionate and made sure I understood everything.”	S2-SDOH
	“My doctor makes me feel comfortable. He’s honest. And I’m honest. I ran into a lot of people that were honest. Nobody just took me as a piece of meat or an experiment or research.”	S1-B
Health system experiences	“The transplant team at IU Health was thorough and really worked with me to navigate everything.”	S1-B
	“I felt at home at the hospital. Whenever I’m here I get the same hospital room, so I’m pretty comfortable. I could wear my own clothes when I was there for my prednisone treatment. Even during the transplant, they made me feel at home.”	S2-SDOH
	“I was in a different city, their program was the point where they said availability was the big problem. H﻿ere in the Midwest and Indianapolis, you have the best chance in the whole country of getting a donor. I was in another state going to a doctor and he said if you want to live, go back to Indiana.”	S2-SDOH
Social support	“I was surrounded by people who cared—my family, my church, even my neighbors helped.”	S2-SDOH
	“I had an﻿ amazing support system from family and friends.”	S2-SDOH
	“I feel great to be around all of y’all so we can think about the person that coming behind﻿ us so we can make a better way for everybody in this moment. And we can be happy in this moment for other people that we’re gonna’ help right now. So cheer up.”	S1-B
Personal growth	“I started valuing my health more, and that led to changes that made a big difference in my life.”	S1-B
	“So I thank God that I have cancer because I was livin’ a drug life as an addict so i﻿f cancer made me stop smokin’ crack or using cocaine or drinkin’…”	S1-B

### Barriers encountered on the journey

#### Systemic issues

Participants discussed various health system challenges, such as frequent changes in doctors, unclear procedures, and insufficient time with medical providers. One patient noted, “I felt lost in the system, like I was just another number. No one really took the time to explain things clearly.” Black patients also noted the lack of minority representation in health care, which influenced their perceptions and experiences. Distrust was a significant issue among Black patients, driven by past experiences of discrimination and skepticism about treatment motives. One participant questioned, “Are they helping me, or are they hurting me?” Another shared concerns about the effects of medication: “I wondered, did they give [cirrhosis] to me [by treating the Hep C]? Because I would’ve probably kept the Hep C instead of having cirrhosis.” Navigating the transplant evaluation phase was daunting for many patients. The complexity and demands of the process, coupled with the uncertainty of the waiting list, added significant stress. As one participant put it, “You have to have all your ducks in a row, or they won’t put you on the list to start with.”

#### Barriers to information

Patients from both groups needed help understanding the vast and often complex medical information provided. As one participant expressed, “You got cancer, here’s a book. I don’t understand that stuff, and I’m still not understanding.” The lack of cohesive information from different providers further compounded this issue: “It’s scattered all over the place.” Both groups reported difficulties communicating with their health care providers. Challenges included trouble reaching providers, delayed test results, and poor communication during hospital stays. One participant highlighted the frustration of accessing test results online before receiving any explanation from a provider: “ …my test was last week. I’ve not heard from anybody up until today… to hear nothing, I start to second guess myself like, did you get my labs?”

#### Experience of treatments and recovery

Patients recounted various physical side effects from their treatments, ranging from common discomforts like fatigue, hair loss, and digestive issues to more severe challenges like intense pain and limited mobility. One participant vividly described their pain: “Two days after [treatment], I felt like I wanted to die. Literally, I wanted to die.” Furthermore, recovery from LT was harder than imagined, and participants described difficulty with daily activities: “Recovery was harder than I imagined. I couldn’t even get out of bed by myself.” The emotional impact of treatment extended to doubts about whether it was worth it: “The medication made me feel horrible. I wasn’t sure if it was worth it.” The emotional toll of recovery was also profound, with patients expressing feelings of depression: “I didn’t expect to feel so depressed during recovery—sometimes I didn’t even want to try anymore.”

#### Social determinants of health

Financial concerns were prominent, including high care costs, insurance issues, and Medicaid limitations: “I was constantly worried about how to pay for my treatment and other bills at the same time.” Lack of social support, particularly among Black patients, was a critical barrier, with one patient revealing, “I didn’t have anyone to help me at home after surgery and that made recovery so much harder.” In addition, living alone made managing treatment difficult: “Living alone made it difficult to manage everything during my treatment. I wish I had someone to rely on.” Finally, transportation was a challenge for patients, particularly in attending appointments: “Getting to all my appointments was a struggle—sometimes I didn’t have the means to get there.”

**FIGURE 2:**
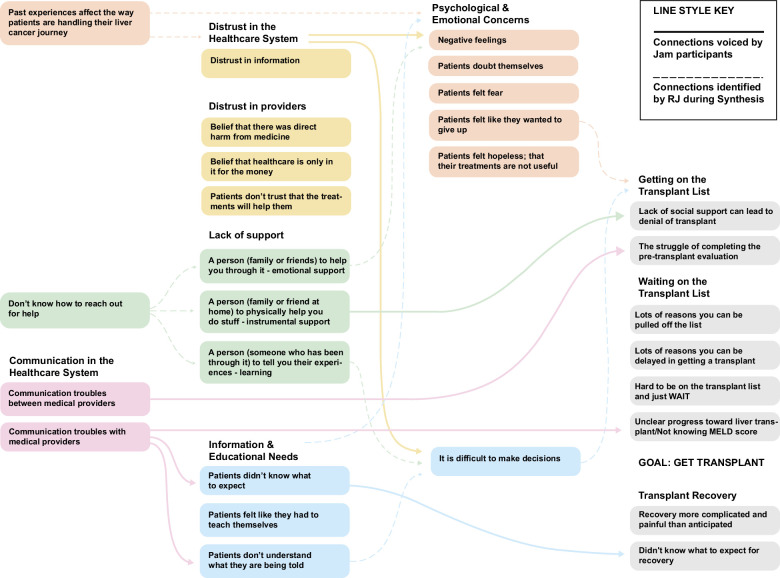
Flow diagram exploring the relationship between challenges.

Table 4 includes additional quotes describing the barriers patients faced on their treatment journey. Figure 2 explores the relationships between the challenges faced by both groups.

**TABLE 4:** Barriers encountered on the journey

Theme	Subtheme	Supporting quotes	Attribution
Systemic issues	Health system issues	“There is poor information sharing between my local health system where my primary care provider is and IU Health”	S2-SDOH
	Distrust in the health care	“I kept questioning whether they were really doing what’s best for me or just following some protocol.”	S1-B
		“I started doubting whether the meds were helping or just making things worse.”	S1-B
	Transplant process challenges	“The process was so complicated, I almost gave up several times because it seemed like I’d never get on the list.”	S1-B
Barriers to information	Information overload	“It was so much information, I felt overwhelmed and couldn’t process it all at once.”	S2-SDOH
	Communication challenges	“It was hard to reach anyone when I had questions—sometimes I had to wait days for a response.”	S1-B
Experience of treatments and recovery	Physical impact of treatment	“The chemo left me so weak, I could barely walk.”	S2-SDOH
	Emotional Impact of Treatment	“The medication made me feel horrible. I wasn’t sure if it was worth it.”	S1-B
		“*I’m just gonna die anyway.”*	S1-B
	Physical challenges in recovery	“Recovery was harder than I imagined. I couldn’t even get out of bed by myself.”	S2-SDOH
	Emotional toll of recovery	“We don’t know how strenuous it is for the recovery process… And it’s like I didn’t want to live anymore. Yes, I was gutted like a fish. And I couldn’t move and I couldn’t stand up and I couldn’t lean to one side. It was uncomfortable… when you lay on your stomach to sleep and you can’t lay on your stomach. You got to pray that you go to sleep, but you can’t sleep on your back… No, I can’t do that. No more.”	S1-B
Social determinants of health	Financial concerns	“I was constantly worried about how to pay for my treatment and other bills at the same time.”	S2-SDOH
	Lack of social support	“I have no social support, especially in terms of after my transplant. No one to come home to help me” “I was denied a transplant because I had no support at home”	S1-B
	Impact of living situation	“I don’t have anywhere to go to or anyone to take care of me after the liver transplant. So I was considering home health care aid, or a nursing home, but it’s like, I couldn’t go there either. So, what is the person supposed to do when they have a liver transplant, but I’m not gonna have anyone which is like…what they…what are they supposed to do when they have no family, no friends, no social support?”	S1-B
	Travel/transportation	“I did the majority of my care at IU Health in Indy [even though I live 45 minutes away] to avoid issues with getting information back and forth”	S2-SDOH

### Group comparisons

**FIGURE 3:**
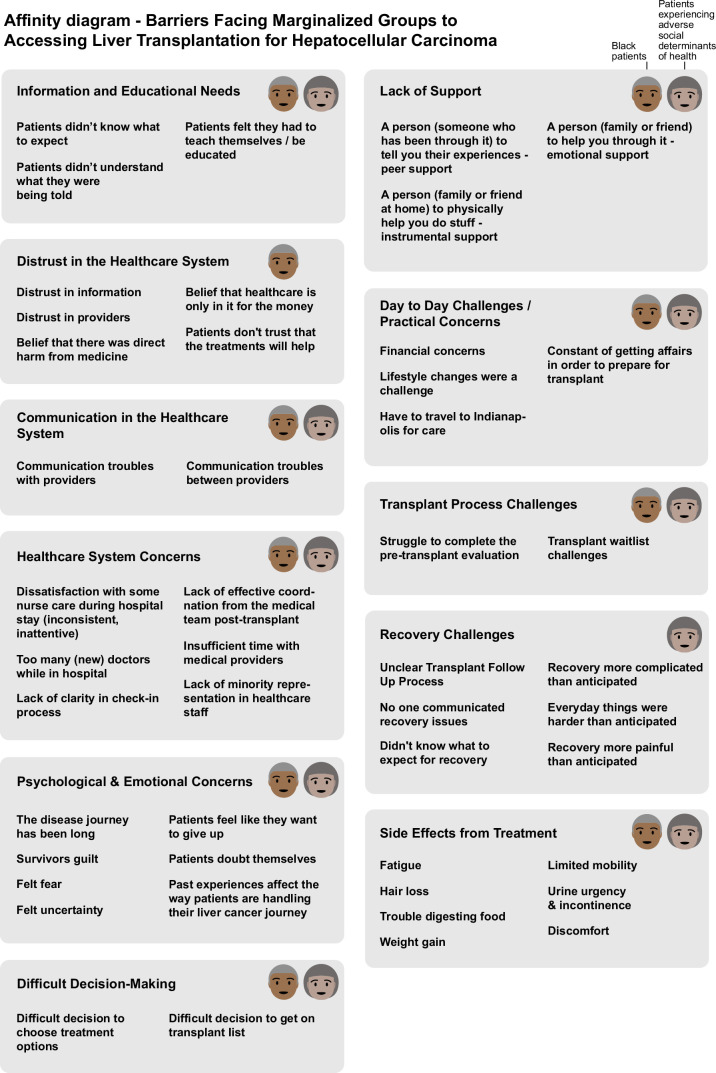
Affinity diagram comparing barriers faced by Black patients and those experiencing adverse social determinants of health.

Both the Black patient group (S1-B) and participants experiencing adverse social determinants of health (S2-SDOH) identified shared challenges such as information overload, difficulties in decision-making, and a strong need for additional peer support. However, distinct differences emerged between the two groups. Black participants in S1-B reported more psychological burdens, including heightened distrust in the health care system and feelings of isolation. In contrast, the S2-SDOH group emphasized logistical and systemic challenges, such as financial concerns, difficulties with care coordination, and travel-related barriers to treatment. Figure 3 summarizes a concept diagram comparing the barriers faced by each group.

### Solutions

Participants provided valuable insights and proposed practical solutions to address the barriers they encountered. These solutions, grounded in their lived experiences, highlight the importance of improving communication, enhancing empathy, providing better preparation, and addressing information gaps. Below, we summarize the proposed solutions along with representative quotes.

#### Clear and consistent communication from providers

Patients emphasized the need for clear and consistent communication from health care providers. Timely updates on test results and treatment progress were seen as essential for reducing anxiety and uncertainty. One participant suggested, “Some type of communication no matter what to say things look good or we need to make this change.”

#### Empathy from health care staff

Participants underscored the importance of empathetic interactions with health care providers. Compassionate care was noted to alleviate distress and foster trust. One participant highlighted this, stating, “Have more empathy from staff for patients. Put yourself in their shoes.” Another added, “Doctors could be more personable with patients, but not overly.”

#### Involvement from care partners and peers

The need for better support and preparation throughout the care continuum was a recurrent theme. Participants recommended involving family members more actively in the treatment plan and connecting patients with peers who had undergone similar treatments. As one participant proposed, “Speaking to a patient who has had the surgery BEFORE surgery—online groups or hospital forums—would help.” Another noted, “A navigator who would be helpful from the beginning to the end, walking you through and explaining things.”

#### Information that is easier to understand

Participants described challenges in understanding medical terminology and managing the information overload associated with their care. They suggested providing simplified, patient-centered educational materials tailored to their needs. One participant remarked, “Help understanding medical terminology would make a big difference.” Another added, “Start talking to patients about recovery early on.”

#### Progress updates while listed

Patients expressed the need for periodic updates about their transplant status and progress, even without specific numerical details. This transparency was viewed as crucial for maintaining hope and engagement. As one participant put it, “A way to indicate progress toward the MELD score—nothing specific in numbers, just a general indication that scores are moving forward—would be helpful.”

**FIGURE 4:**
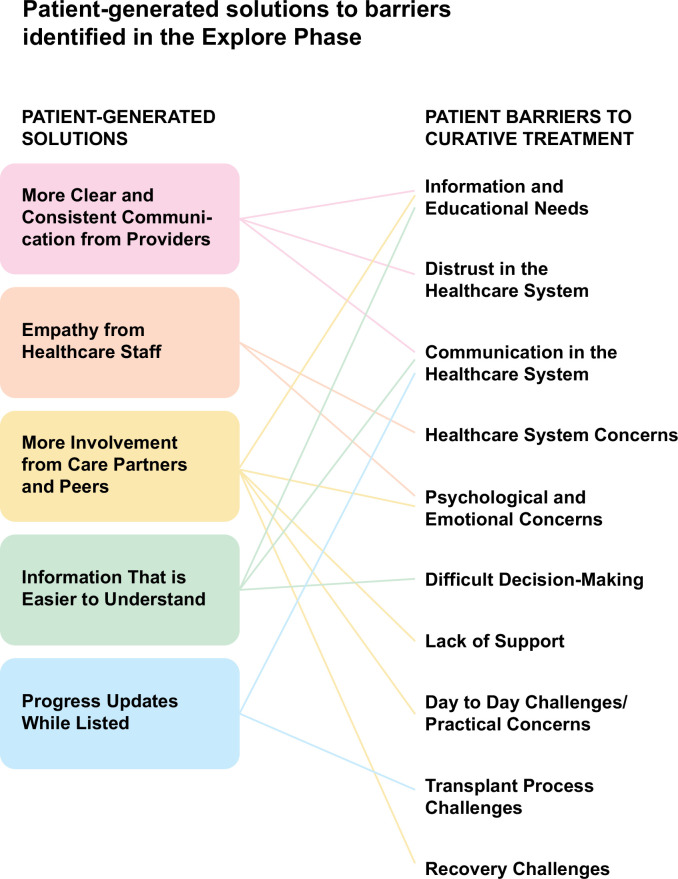
Patient provided solutions to barriers identified to accessing curative therapies.

Figure 4 maps solutions suggested by patients onto challenges identified by both groups.

## DISCUSSION

The study results reveal a nuanced understanding of the experiences of patients seeking curative HCC therapies. Marginalized groups encounter multifaceted challenges in accessing curative therapies for HCC, including information overload, lack of instrumental and peer support, and distrust in health care. This did not mitigate their impressions about the positive impact of supportive patient-provider relationships and the significant instrumental and emotional support provided by family and friends. While both groups described many similar barriers, the unique differences may be crucial to informing future interventions.

One of the standout themes in the study was the positive impact of supportive provider-patient relationships. Participants consistently highlighted the importance of compassionate and knowledgeable health care providers who were crucial in their journey. These relationships were characterized by empathy, trust, and effective communication, which were instrumental in alleviating anxiety and fostering a sense of security during a challenging medical process. This finding is consistent with other qualitative literature on patients undergoing LT evaluation that found communication between patients and transplant providers is an important dimension of patient engagement during evaluation.^14^ Participants generally reported high levels of satisfaction with the care received within the IU Health system. For some, the multidisciplinary nature of care provided reassurance and confidence in the treatment plan, contributing positively to their overall experience. While the positive impact of patient-provider relationships on patient outcomes has not been studied in liver disease or HCC, the effect on overall health and cancer outcomes has been explored. A systematic review and meta-analysis of randomized controlled trials found that positive patient–clinician relationships are associated with objective (eg, survival rates, disease progression) and validated subjective (eg, patient-reported quality of life) outcome measures.^15^ Strong patient-provider relationships also help address cancer patients’ psychological and emotional needs.^16^ Fostering trust through transparent, empathetic communication can reduce barriers to care for marginalized communities, promoting better engagement and access to essential health services.^17^

The study highlighted the significant role of social support networks, including family, friends, and peers in the patient experience. Participants described how emotional and instrumental support from loved ones helped them navigate challenges such as treatment decisions, recovery, and lifestyle adjustments after the transplant. For some, social support acted as a buffer against stress and isolation, enhancing resilience and well-being during a critical period of their lives. In contrast, many participants, particularly Black patients, reported challenges associated with inadequate social support networks. This lack of support exacerbated feelings of isolation and emotional distress, impacting their ability to cope with the demands of HCC treatment, including LT. Addressing these disparities in social support is critical to improving the overall experience and outcomes of Black patients. In HCC and end-stage liver disease, marital or partnered status, often used as a surrogate for social support in large data sets that lack more explicit data about the construct, has been explored and associated with survival. A study by Kroenke et al^18^ found that women with colorectal cancer who had low social support before diagnosis had a 42% higher mortality rate than those with high levels of support. Furthermore, this is an intervenable construct. A meta-analyses of psychosocial support interventions, including support groups and individual counseling, have shown that these interventions can improve patient survival and quality of life.^19^

Despite the positive aspects of provider relationships, participants often faced challenges related to information overload and communication gaps. Many patients felt overwhelmed by the volume and complexity of medical information provided, which sometimes led to confusion and difficulties in decision-making. Moreover, some participants highlighted instances where communication breakdowns between health care providers and patients occurred, resulting in misunderstandings and unmet information needs. Unmet informational needs have been explored in chronic liver disease and cancer types.^20^,^21^,^22^ In a study of patients with colorectal cancer, significant unmet informational needs, especially regarding treatment side effects, psychosocial impacts, and long-term management were identified.^23^ Furthermore, in autoimmune hepatitis, there was a higher burden of unmet informational needs among participants with low socioeconomic status.^22^ These unmet needs can lead to increased anxiety, reduced treatment adherence, and poorer quality of life. However, patients’ feelings of informational overload and the need for more information pose a paradox. Interventions in this space must provide information on what patients want to know (why, how we treat, side effects, and what to expect after treatment) in manageable, understandable doses. This includes employing precise, jargon-free communication techniques and multimedia tools to enhance understanding of complex medical information. Moreover, educational initiatives should promote the active involvement of patients in shared decision-making processes, equipping them with the knowledge and confidence to participate effectively in their care.^24^ By fostering health literacy and patient empowerment, educational efforts can promote informed decision-making, improve treatment adherence, and ultimately optimize outcomes for patients undergoing liver cancer treatment, LT, or resection.

A notable finding was the presence of distrust in the health care system among Black patients. Historical and current experiences of discrimination, systemic biases, and unmet informational needs likely contributed to skepticism about medical recommendations and treatment outcomes.^23^ This distrust can influence health care-seeking behaviors and patient-provider interactions, posing barriers to effective care delivery and patient satisfaction.^25^ Health care system distrust in breast cancer led to delays in seeking treatment, lower rates of adherence to treatment plans, and overall poorer health outcomes.^26^,^27^ Interventions to improve medical distrust have focused on enhancing patient-provider communication, transparency, community engagement, and cultural competence training.^26^,^28^ Recently, virtual environments that facilitate those constructs have also been employed.^29^

Finally, this study contributes to the growing body of literature highlighting the critical role of patient input in designing interventions for chronic liver disease, particularly among marginalized populations.^30^,^31^,^32^ While limited research has applied these principles to liver disease care, this approach enhances the relevance of interventions and fosters trust and engagement—key elements in managing complex conditions like HCC. The participant-proposed solutions further underscore the importance of patient-centered care, emphasizing consistent communication and empathetic interactions as essential for building trust and reducing distress. Practical strategies such as navigators, peer support, and progress updates during the transplant process address both informational and emotional challenges, aligning with evidence-supporting patient-centered approaches in improving care delivery.

This study is the first to explore the barriers experienced by marginalized groups and the solutions they proposed to overcome them in seeking curative therapies for HCC. The HCD methodology enabled an in-depth exploration of patient experiences across various stages of the care continuum capturing rich, nuanced data that provided valuable insights into the perspectives of patient groups experiencing significant disparities in HCC-related outcomes. Visual modeling and affinity diagramming facilitated the identification of common themes and patterns, ensuring comprehensive coverage of both positive and negative aspects of the treatment journey as well as barriers encountered by patients. This deep contextual reporting allows readers to assess the relevance of these findings to their settings and provides rich data to inform intervention development in settings outside of IUH. Additionally, while differences between the 2 groups were evident, our study was limited by having only 2 Black patients who had received LT and experienced the entire care continuum. While this did not hinder our understanding of the challenges faced by other Black patients aiming for a cure, it left us with limited data on Black patients’ experiences related to waiting for LT and post-LT recovery. Future multicenter studies are needed to capture Black patients’ experiences at each step in the care continuum for both LT and resection. Despite these limitations, the study offers valuable insights into the lived experiences of patients with HCC, highlighting avenues for future research and health care improvements.

In conclusion, the analysis underscores the multifaceted nature of the patient experience within the HCC-curative therapy continuum of care. While positive provider relationships, quality of care, and social support networks contribute positively to patient well-being, information overload, communication barriers, distrust in health care, and disparate social support resources require further investigation in order to develop effective interventions. Furthermore, while marginalized groups may all face significant obstacles in accessing curative therapies, the nature of these challenges varies, highlighting the need for tailored interventions. Finally, patients understand their unmet needs and can offer pragmatic solutions. Failure to actively engage patients during the development of interventions that are misaligned with their priorities and needs potentially leads to inefficient use of resources and suboptimal outcomes. Future research and transplant programs should consider these lessons as they strive to improve quality and equity.

## Supplementary Material

## Data Availability

Interview guide available on reasonable request. This work was supported by NIMHD 1K23MD018090-01, Indiana University Health Equity Advancing through Learning Health System Research (HEAL-R) Collaborative Award, NIDDK K08DK133638. Nicole Garcia is employed by the Indiana University School of Medicine. The remaining authors have no conflicts to report. The institutional review board approved the study. All participants provided written informed consent to participate in the study.

## References

[1] Adra S Alabrach Y Hashem A Mahmoud A Khalouf A El-Khapery A . Trends of primary liver cancer incidence and mortality in the United States: A population-based study over the last four decades. PLoS One. 2024;19:e0309465. 39236039 10.1371/journal.pone.0309465 PMC11376511

[2] Chakraborty E Sarkar D . Emerging therapies for hepatocellular carcinoma (HCC). Cancers. 2022;14:2798. 35681776 10.3390/cancers14112798 PMC9179883

[3] Nephew LD Serper M . Racial, gender, and socioeconomic disparities in liver transplantation. Liver Transpl. 2021;27:900–912. 33492795 10.1002/lt.25996

[4] Siegel AB McBride RB El-Serag HB Hershman DL Brown RS Renz JF . Racial disparities in utilization of liver transplantation for hepatocellular carcinoma in the United States, 1998–2002. Am J Gastroenterol. 2008;103:120–127. 18005365 10.1111/j.1572-0241.2007.01634.x

[5] Nephew LD Gupta D Carter A Desai AP Ghabril M Patidar KR . Social determinants of health impact mortality from HCC and cholangiocarcinoma: a population-based cohort study. Hepatol Commun. 2023;7:e0058. 36757397 10.1097/HC9.0000000000000058 PMC9916098

[6] Nephew LD Aitcheson G Iyengar M . The impact of racial disparities on liver disease access and outcomes. Curr Treat Options Gastroenterol. 2022;20:1–16.

[7] Glenn J Kleinhenz G Smith JMS Chaney RA Moxley VBA Donoso Naranjo PG . Do healthcare providers consider the social determinants of health? Results from a nationwide cross-sectional study in the United States. BMC Health Serv Res. 2024;24:271. 38438936 10.1186/s12913-024-10656-2 PMC10910743

[8] Teisberg E Wallace S O’Hara S . Defining and implementing value-based health care: A strategic framework. Acad Med. 2020;95:682–685. 31833857 10.1097/ACM.0000000000003122 PMC7185050

[9] Hundt M Chen A Donovan J Kim N Yilma M Tana M . Barriers to liver transplant referral in safety net settings: A national provider survey. Liver Transpl. 2024;30:896–906. 38687168 10.1097/LVT.0000000000000384

[10] Leung CL Naert M Andama B Dong R Edelman D Horowitz C . Human-centered design as a guide to intervention planning for non-communicable diseases: The BIGPIC study from Western Kenya. BMC Health Serv Res. 2020;20:415. 32398131 10.1186/s12913-020-05199-1 PMC7218487

[11] Tucker Edmonds B Hoffman SM Lynch D Jeffries E Jenkins K Wiehe S . Creation of a decision support tool for expectant parents facing threatened periviable delivery: Application of a user-centered design approach. Patient. 2019;12:327–337. 30488236 10.1007/s40271-018-0348-y PMC6686668

[12] Brest P Roumani N Bade J . Problem solving, human-centered design, and strategic processes. In: Conference Paper, Vol. 205. 2015:15. Docx. Accessed November_1_2024 http://Pacscenter.Stanford.Edu/Sites/All/Files/Brest%20Roumani%20Bade%20Solving%20and%20Strategic%20Processes%20v%2015a

[13] Brown T . Change by Design: How Design Thinking Transforms Organizations and Inspires Innovation, 1st ed. HarperCollins Publishers; 2009.

[14] Strauss AT Brundage J Sidoti CN Jain V Gurakar A Mohr K . Patient perspectives on liver transplant evaluation: A qualitative study. Patient Educ Couns. 2024;127:108346. 38896893 10.1016/j.pec.2024.108346 PMC11323235

[15] Kelley JM Kraft-Todd G Schapira L Kossowsky J Riess H . The influence of the patient–clinician relationship on healthcare outcomes: A systematic review and meta-analysis of randomized controlled trials. PLoS One. 2014;9:e94207. 24718585 10.1371/journal.pone.0094207 PMC3981763

[16] Smith K.L Martini J . Patient-Provider Communication and Interactions. In: Daaleman, TP, Helton, MR, eds. Chronic Illness Care. Springer, Cham; 2023. 10.1007/978-3-031-29171-5_14

[17] Griffith DM Bergner EM Fair AS Wilkins CH . Using mistrust, distrust, and low trust precisely in medical care and medical research advances health equity. Am J Prev Med. 2021;60:442–445. 33208267 10.1016/j.amepre.2020.08.019 PMC7902381

[18] Kroenke CH Paskett ED Cené CW Caan BJ Luo J Shadyab AH . Prediagnosis social support, social integration, living status, and colorectal cancer mortality in postmenopausal women from the women’s health initiative. Cancer. 2020;126:1766–1775. 31972054 10.1002/cncr.32710 PMC7297047

[19] Smith TB Workman C Andrews C Barton B Cook M Layton R . Effects of psychosocial support interventions on survival in inpatient and outpatient healthcare settings: A meta-analysis of 106 randomized controlled trials. PLoS Med. 2021;18:e1003595. 34003832 10.1371/journal.pmed.1003595 PMC8130925

[20] Lu H Xie J Gerido LH Cheng Y Chen Y Sun L . Information needs of breast cancer patients: Theory-generating meta-synthesis. J Med Internet Res. 2020;22:e17907. 32720899 10.2196/17907 PMC7420822

[21] Abu Sharour L Malak M Subih M Bani Salameh A . Quality of life, care needs, and information needs among patients diagnosed with cancer during their treatment phase. Psychol Health Med. 2020;25:252–258. 31795738 10.1080/13548506.2019.1699660

[22] Singleton C Carter A Baker B Jones E Green K Lammert C . Low socioeconomic status exacerbates unmet health-related needs in patients with autoimmune hepatitis. Aliment Pharmacol Ther. 2024;60:1339–1350. 39254160 10.1111/apt.18235

[23] Dau H Safari A Saad El Din K McTaggart-Cowan H Loree JM Gill S . Assessing how health information needs of individuals with colorectal cancer are met across the care continuum: An international cross-sectional survey. BMC Cancer. 2020;20:1031. 33109114 10.1186/s12885-020-07539-0 PMC7590465

[24] Reyes KR Wong P Petrofsky M Dai A Pelayo A Brondfield S . Shared decision-making needs, barriers, and facilitators of patients with newly diagnosed advanced cancer in the hospital: A multi-level, mixed-methods study. Support Care Cancer. 2024;32:315. 38684522 10.1007/s00520-024-08515-1 PMC11058864

[25] Thompson VL Li Y Liu Y Hong J Sharma S Metoyer G . Medical distrust among kidney transplant candidates. Clin Transplant. 2024;38:e15395. 39023087 10.1111/ctr.15395 PMC11259129

[26] Mouslim MC Johnson RM Dean LT . Healthcare system distrust and the breast cancer continuum of care. Breast Cancer Res Treat. 2020;180:33–44. 31983018 10.1007/s10549-020-05538-0 PMC7675785

[27] Wilder JM Oloruntoba OO Muir AJ Moylan CA . Role of patient factors, preferences, and distrust in health care and access to liver transplantation and organ donation. Liver Transpl. 2016;22:895–905. 27027394 10.1002/lt.24452 PMC5567682

[28] Ledford CJW Villagran MM Kreps GL Zhao X McHorney C Weathers M . “Practicing medicine”: Patient perceptions of physician communication and the process of prescription. Patient Educ Couns. 2010;80:384–392. 20675096 10.1016/j.pec.2010.06.033

[29] Ramos SR Warren R Shedlin M Melkus G Kershaw T Vorderstrasse A . A framework for using eHealth interventions to overcome medical mistrust among sexual minority men of color living with chronic conditions. Behav Med. 2019;45:166–176. 31343963 10.1080/08964289.2019.1570074 PMC6793989

[30] Paradise RK Dryden E Elvin D Fisher C Touw S Trumble L . Incorporating patient input into the design of a disease management program for COPD. Healthc. 2020;8:100363. 10.1016/j.hjdsi.2019.05.003 31147276

[31] Hudon C Fortin M Haggerty J Loignon C Lambert M Poitras ME . Patient-centered care in chronic disease management: A thematic analysis of the literature in family medicine. Patient Educ Couns. 2012;88:170–176. 22360841 10.1016/j.pec.2012.01.009

[32] Alcaraz KI Yanez BR . Interventions to promote health equity: Implications for implementation science in behavioral medicine. Transl Behav Med. 2022;12:885–888. 36205475 10.1093/tbm/ibac062 PMC9540972

