# Supplementary material for: Information overload, financial constraints, and psychological burdens are among the barriers faced by marginalized groups seeking curative treatments for HCC
Source: Hepatol Commun. 2025 Feb 26;9(3):e0660. doi: 10.1097/HC9.0000000000000660 (PMC11868430; doi:10.1097/HC9.0000000000000660)

**Supplemental Figure 1: Session Activities**

Panel A: Challenge category sheet

Panel B: Ideation prompt list

Panel C: Group journey map


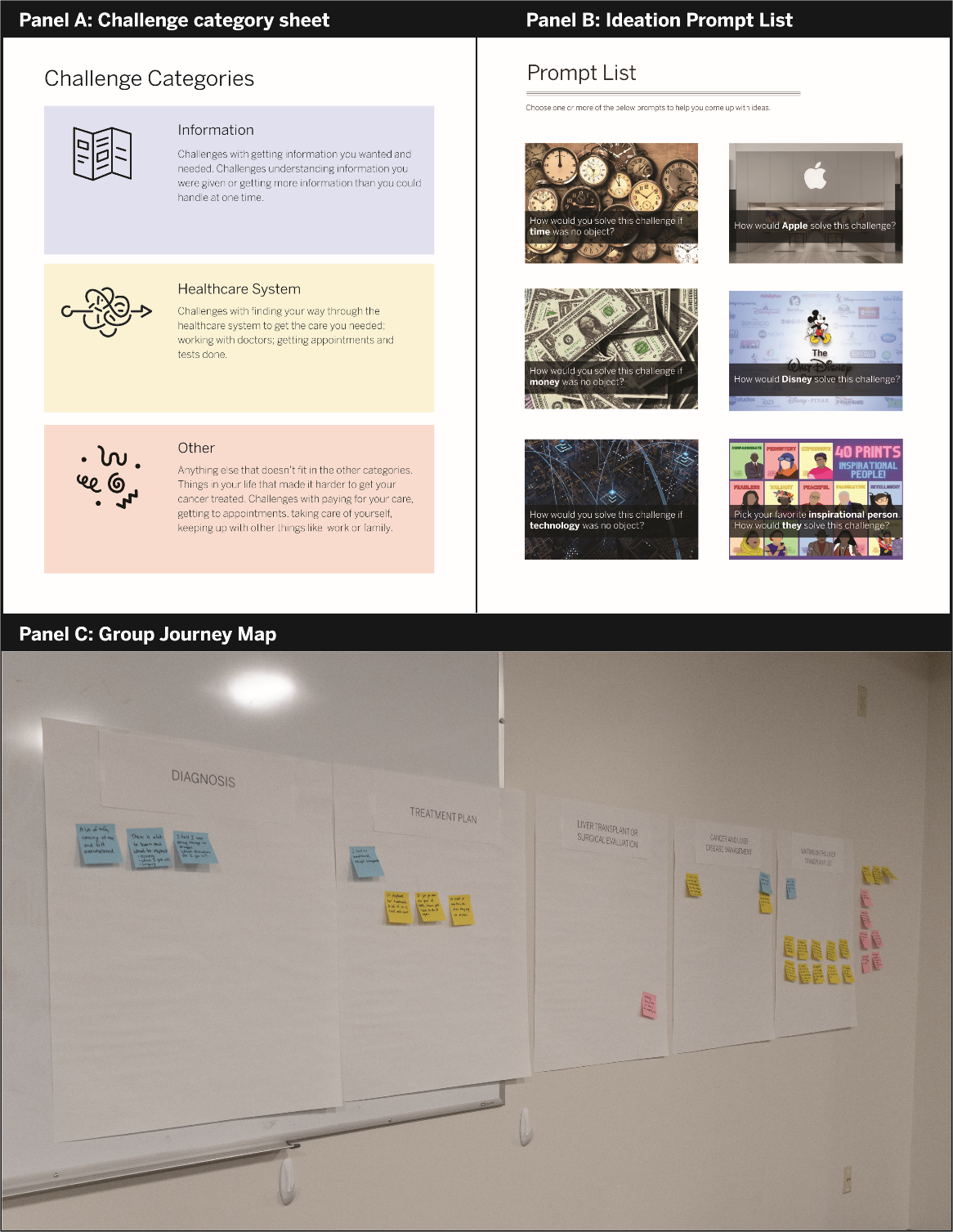

Supplement: Supplementary file 1 [file hc9-9-e0660-s001.docx]
