# Supplementary material for: Information overload, financial constraints, and psychological burdens are among the barriers faced by marginalized groups seeking curative treatments for HCC
Source: Hepatol Commun. 2025 Feb 26;9(3):e0660. doi: 10.1097/HC9.0000000000000660 (PMC11868430; doi:10.1097/HC9.0000000000000660)
Supplement: Supplementary file 2 [file hc9-9-e0660-s002.docx]

**Supplemental Table 1: One-on-One Interview Questions**

| **Primary Goal/Concentration** | **Probing Questions** |
| --- | --- |
| **Warm-Up** | - Pick one card and answer the question on it:   - If you had to teach a class on one thing, what would you teach?   - If you could instantly become an expert in something, what would it be?   - If you could have any superpower what would it be and why?   - If you could go to Mars, would you? Why or why not?   - What’s your favorite place in the world, that is not your house? |
| **Journey Map & Challenges** | - Can you describe how you have felt throughout this process? - Tell me about your challenges with your liver cancer journey. What are your biggest challenges that stand out to you. |
| **Deeper Dive** | - Were you ever unclear of your progress toward liver transplant? Not knowing MELD score? If so, can you tell me about it? - Did you struggle to complete the transplant evaluation? How so? - Was it difficult to get stuff done to prepare for your treatments/transplant/recovery? - What was the hardest part about being on the transplant waitlist - Do you feel like you had enough support? If not, tell me more. - Was there ever a time when you were unsure of what your doctors were recommending for you? Why or why not? - Did you have challenges dealing with all the information given to you about liver cancer? If so, what challenges did you have? - How satisfied were you with your hospital stay? - What was your recovery like? - Did you have any lifestyle changes you had to make? - Have there been any resources that have been really helpful to you? If so, what are they? (Support groups, social workers, Facebook groups, Little Red Door) - Was there anything that surprised you about this journey? - What was your least favorite part about the whole liver cancer journey? - What was the best part of this experience (or was there something positive about this experience...that you think every patient should have...)? |
| **Ideas you have** | - If you have one suggestion for us to make this experience better, what would that be? |
